# Inhibition of protein geranylgeranylation induces apoptosis in synovial fibroblasts

**DOI:** 10.1186/ar1968

**Published:** 2006-06-14

**Authors:** Alison M Connor, Stuart Berger, Aru Narendran, Edward C Keystone

**Affiliations:** 1 The Wellesley Toronto Arthritis and Immune Disorder Research Centre, 101 College St. Toronto, Ontario, Canada M5G 1L7; 2 Southern Alberta Children's Cancer Program, Alberta Children's Hospital, 1820 Richmond Road SW Calgary, Alberta, Canada T2T 5C7; 3 The Rebecca MacDonald Centre for Arthritis and Autoimmune Disease, Mount Sinai Hospital, 60 Murray Street, Toronto, Ontario, Canada, M5T 3L9

## Abstract

Statins, competitive inhibitors of hydroxymethylglutaryl-CoA reductase, have recently been shown to have a therapeutic effect in rheumatoid arthritis (RA). In RA, synovial fibroblasts in the synovial lining, are believed to be particularly important in the pathogenesis of disease because they recruit leukocytes into the synovium and secrete angiogenesis-promoting molecules and proteases that degrade extracellular matrix. In this study, we show a marked reduction in RA synovial fibroblast survival through the induction of apoptosis when the cells were cultured with statins. Simvastatin was more effective in RA synovial fibroblasts than atorvastatin, and both statins were more potent on tumor necrosis factor-α-induced cells. In contrast, in osteoarthritis synovial fibroblasts, neither the statin nor the activation state of the cell contributed to the efficacy of apoptosis induction. Viability of statin-treated cells could be rescued by geranylgeraniol but not by farnesol, suggesting a requirement for a geranylgeranylated protein for synovial fibroblast survival. Phase partitioning experiments confirmed that in the presence of statin, geranylgeranylated proteins are redistributed to the cytoplasm. siRNA experiments demonstrated a role for Rac1 in synovial fibroblast survival. Western blotting showed that the activated phosphorylated form of Akt, a protein previously implicated in RA synovial fibroblast survival, was decreased by about 75%. The results presented in this study lend further support to the importance of elevated pAkt levels to RA synovial fibroblast survival and suggest that statins might have a beneficial role in reducing the aberrant pAkt levels in patients with RA. The results may also partly explain the therapeutic effect of atorvastatin in patients with RA.

## Introduction

Rheumatoid arthritis (RA) is a chronic inflammatory disease causing progressive joint destruction, deformity and disability. The pathogenesis of the rheumatoid joint involves hyperplasia of the synovial lining cells, mononuclear cell infiltration and new blood vessel formation within the synovium as well as the destruction of cartilage and underlying bone as a consequence of pro-inflammatory cytokines and proteases [1]. Much of the pathology is thought to be driven by cytokines, particularly tumor necrosis factor α (TNF-α) [2].

Synovial tissue consists primarily of two distinct cell types: the macrophage-like synoviocytes and synovial fibroblasts. The synovial fibroblasts are important in all aspects of the pathogenesis of arthritis. Hyperplasia of the synovial lining in RA is due primarily to increases in the number of synovial fibroblasts. Although the reason for this increase is currently unknown, impaired apoptosis or senescence has been proposed to explain their increased numbers [3].

The RA synovial fibroblast response to the macrophage-derived cytokines TNF-α and IL-1 includes elevated expression of adhesion molecules, cytokines and chemokines. RA synovial fibroblasts also secrete angiogenesis-promoting molecules such as vascular endothelial growth factor A and several proteases, including matrix metalloproteinases, aggrecanases and cathepsins, that mediate extracellular matrix degradation [4].

TNF-α is capable of signaling both cell-survival and cell-death signals. The response of a cell to TNF-α depends on specific adaptors and downstream signaling molecules [5]. The addition of TNF-α to RA synovial fibroblasts results in resistance to apoptosis and hence to increased survival as well as proliferation [6]. Recent reports have indicated that it is possible to reverse the survival response of RA synovial fibroblasts to TNF-α by inhibiting the translocation of nuclear factor κB to the nucleus [7], or ectopically expressing TIMP (tissue inhibitor of metalloproteinases) 3 [8]. The ability to reverse resistance of fibroblast-like synoviocytes (FLS) to apoptosis could represent an important therapeutic target in arthritis [9].

Statins, competitive inhibitors of hydroxymethylglutaryl (HMG)-CoA reductase, were initially designed as inhibitors of cholesterol synthesis [10]. HMG-CoA reductase catalyzes the conversion of HMG-CoA to mevalonate, a rate-limiting step in cholesterol biosynthesis. However, statins seem to have anti-inflammatory effects that cannot be accounted for by their lipid-lowering abilities. These include the suppression of proinflammatory cytokine and chemokine production, immunomodulation and the downregulation of endothelial cell activation [11,12]. As a consequence of these properties, statin therapy has been examined in several chronic immune-mediated inflammatory diseases including experimental autoimmune encephalomyelitis and arthritis. The statin simvastatin has been shown to exhibit a therapeutic effect in the collagen-induced arthritis (CIA) model of RA [13]. It was thought to exert its effect through decreasing the viability of T helper type 1 cells and attenuating the interaction of T cells with macrophages. In contrast with these results, another study showed that neither atorvastatin nor rosurvastatin had a beneficial effect on the mouse CIA model of arthritis. The results of simvastatin could be accounted for by severe side effects [14]. Nevertheless, atorvastatin was found to have a therapeutic effect in patients with RA as well as beneficially influencing inflammatory markers [15].

Some of the beneficial effects of statins can be explained by the fact that the inhibition of mevalonate synthesis by statins also prevents the synthesis of other intermediates along the cholesterol biosynthetic pathway. These include the isoprenoid intermediates geranylgeranylpyrophosphate (GGPP) and farnesylpyrophosphate (FPP), which are required for the prenylation of many cellular proteins such as the small GTP-binding proteins Ras, Rho and Rac [16]. Isoprenylation enables the insertion of these proteins into the cellular membrane, where they function in intracellular signal transduction pathways [17]. It is significant that statins have also been shown to induce apoptosis, mediated by inhibition of the synthesis of FPP and GGPP [18-20].

Because reduced apoptosis of synovial fibroblasts in RA is thought to be a cause of synovial hyperplasia, statins could theoretically reduce joint damage by interfering with synovial fibroblast survival. The aim of the present study was therefore to determine whether statins could substantially reduce RA synovial fibroblast survival and whether one statin would be particularly effective. Our data suggest that simvastatin is significantly more effective than atorvastatin at decreasing the viability of RA synovial fibroblasts, particularly in the presence of TNF-α. This contrasts with the viability of synovial fibroblasts derived from patients with osteoarthritis (OA), in whom no differential effect of the statins was observed. The decreased viability of RA synovial fibroblasts with statins was shown to be mediated by the inhibition of membrane-associated geranylgeranylated proteins and was associated with decreased pAkt levels.

## Materials and methods

### Synovial tissue

The protocol for patient consent and the use of human tissues was approved by ethics review committees at both the University Health Network and St Michael's Hospital. All tissue was obtained with patient consent.

### Cell culture

Synovial fibroblasts were isolated from synovial tissues of patients with RA or OA removed at the time of arthroplasty, as described previously [21]. Cells were maintained in Opti-MEM supplemented with 4% fetal bovine serum and 1% antibiotic-antimycotic (Life Technologies, Rockville, MD, USA) and were cultured at 37°C in a humidified chamber containing 95% air, 5% CO_2_. Synovial fibroblasts were used at passages 2 to 4. Unless indicated otherwise, cells used for Western blots were plated at 10^5 ^cells per well in a six-well plate for 48 hours before initiation of experiments.

### Effect of statins on cellular viability

Stock solutions of lovastatin (Sigma-Aldrich, Oakville, Ontario, Canada) and atorvastatin (Toronto Research Chemicals, North York, Ontario, Canada) were prepared in dimethylsulfoxide (DMSO). Cerivastatin (Toronto Research Chemicals) was prepared in PBS and simvastatin (Calbiochem, La Jolla, CA, USA) was activated and neutralized in accordance with the manufacturer's directions. Recombinant human TNF-α was purchased from R&D Systems Inc. (Minneapolis, MN, USA). GGPP and FPP, sodium 3-(1-(phenylamino-carbonyl)-3, 4-tetrazolium)-bis (4-methoxy-6-nitro) benzenesulfonic acid hydrate (XTT), and phenazine methosulfate (PMS) were obtained from Sigma-Aldrich. Synovial fibroblasts (*n *= 6 RA, *n *= 6 OA) were plated in 100 μl at a concentration of 3 × 10^4 ^cells/ml in a 96-well plate. Before treatment with statin or carrier control, they were cultured for 24 hours in the presence or absence of 10 ng/ml TNF-α. Statins were then added to the wells and cells were continued to be cultured for the duration indicated in the figure legends. Where indicated, synovial fibroblast cultures were supplemented with 5 μM FPP or 5 μM GGPP at the time of statin addition [22]. Cellular viability was determined by the XTT assay on quadruplicate wells [23]. XTT (1 mg/ml; 50 μl) and 1 μl of 1.25 mmol (PMS) were added to each well and plates were cultured for a further 4 hours. Colour development was measured at 490 nm with a reference wavelength of 650 nm.

### Phase partitioning

RA synovial fibroblasts were incubated for 72 hours with 3 μM simvastatin in the presence or absence of 5 μM FPP or 5 μM GGPP. Integral membrane proteins were extracted by Triton X-114 as described [24]. In brief, 200 μl of lysis solution (10 mM Tris-HCl pH 7.4, 150 mM NaCl, 1.0% Triton X-114) was added to each well of a six-well culture plate and incubated on ice for 30 minutes. Samples were scraped from the plate and overlaid on a sucrose cushion. After a 3-minute incubation at 30°C they were centrifuged at 300 *g *for 3 minutes to separate the phases. The upper aqueous phase was rinsed twice; then Triton X-114 and buffer were added to the aqueous and detergent phases, respectively, to yield equal volumes and approximately the same salt and detergent concentration in both fractions. Samples were separated on a 12% SDS-polyacrylamide gel. Fractionated proteins were transferred to a poly(vinylidene difluoride) membrane (Immobilon-P; Millipore Corp, Bedford, MA, USA). Membranes were blocked for 1 hour in blocking buffer (10 mM Tris pH 7.5, 2.5 mM EDTA, 100 mM NaCl, 0.1% Tween 20) containing 3% gelatin, and then probed with anti-RhoA (Santa Cruz Biotechnology Inc., Santa Cruz, CA, USA) or anti-Rac1 (BD Biosciences, Mississauga, Ontario, Canada) for 1 hour. After being washed, membranes were incubated with a goat anti-mouse IgG conjugated to horseradish peroxidase secondary antibody (Sigma-Aldrich) for a further 1 hour, and bands were revealed by enhanced chemiluminescence (ECL; Amersham Pharmacia Biotech, Baie d'Urfe, Québec, Canada).

### Cell Death Detection ELISA

Cytoplasmic histone-associated DNA fragments associated with apoptotic cell death were determined quantitatively with the Cell Death Detection ELISA^PLUS ^(Roche Molecular Biochemicals, Mannheim, Germany). Cells were plated at 1.5 × 10^4 ^cells per well in a 24-well plate and treated for 72 hours with DMSO or with 3 or 10 μM lovastatin in the presence or absence of 5 μM FPP or 5 μM GGPP as indicated. Cells were lysed with 200 μl of lysis buffer; 20 μl was then added to the microtiter plate followed by 80 μl of immunoreagent. After incubation for 2 hours at room temperature, the plate was washed and developed with ABTS (2,2'-azino-bis(3-ethylbenzthiazoline-6-sulfonic acid)) solution. Each experimental condition was performed in duplicate.

### TUNEL (TdT-mediated dUTP nick end labelling) staining of synovial fibroblasts

RA synovial fibroblast apoptotic nuclei were detected by a TdT-FragEL DNA fragmentation kit in accordance with the manufacturer's directions (Oncogene Research Products, Cambridge, MA, USA). Cells were plated at 2 × 10^4 ^per chamber of a two-well chamber slide and treated for 72 hours with DMSO or 10 μM lovastatin in the presence or absence of 5 μM FPP or 5 μM GGPP as indicated in the figure legends. After TUNEL staining, cells were counterstained with hematoxylin. This was performed on two different lines.

### Analysis of phosphorylated proteins

RA synovial fibroblasts were treated with DMSO or 3 μM lovastatin for 48 hours and subsequently cultured for 15 minutes with 10 ng/ml TNF-α or 2 ng/ml IL-1 (R&D Systems Inc, Minneapolis, MN, USA). Cells were then rinsed twice with PBS and lysed in 20 mM Tris pH 7.5, 150 mM NaCl, 1 mM EDTA, 1 mM EGTA, 1% Triton X-100, 2.5 mM sodium pyrophosphate, 1 mM β-glycerolphosphate, 0.5 μg/ml leupeptin, 0.2 mM phenylmethylsulphonyl fluoride. Protein concentrations were determined by the micro bicinchoninic acid (BCA) protein assay kit (Pierce, Rockford, IL, USA) to enable comparable sample loading on a 12% SDS-polyacrylamide gel. SDS-PAGE was performed as described for the phase partitioning. pJNK (Thr183/Tyr185), pERK and pAkt (Ser473) antibodies were obtained from Cell Signaling Technology (Beverly, MA, USA). Prehybridization and hybridization were performed in accordance with the manufacturer's protocol. Blots were scanned and band intensities were measured with the UN-SCAN-IT version 5.1 software (Silk Scientific, Orem, UT, USA). Relative amounts of protein were expressed as multiples of the solvent control.

Blots were stripped between probings with Re-Blot-Plus (Chemicon International, Inc., Temecula, CA, USA).

### siRNA

To confirm the involvement of geranylgeranylated proteins in FLS survival and pAkt expression, siRNA for Rac1 (target sequence 5'-AACCGGTGAATCTGGGCTTAT-3') was transfected into RA synovial fibroblasts (*n *= 3) plated at 6.0 × 10^4 ^cells per well of a six-well plate or 3 × 10^3 ^cells per well of a 96-well plate, at an siRNA to RNAiFect Reagent (Qiagen Inc., Mississauga, Ontario, Canada) ratio of 1:4.5. In preliminary experiments, a non-silencing control siRNA labeled with Alexa Fluor 488 was monitored by fluorescence microscopy. The conditions used in this study resulted in a cell transfection efficiency of greater than 95%. The medium was changed 18 hours after transfection. XTT assays were performed and cell lysates were prepared 96 hours after transfection as described above.

### Statistics

Results are presented as means ± SD. Significant differences between groups were analysed by using a two-tailed unpaired Student *t *test; *p *< 0.05 was considered statistically significant.

## Results

### Statins decrease synovial fibroblast viability

**Figure 1:**
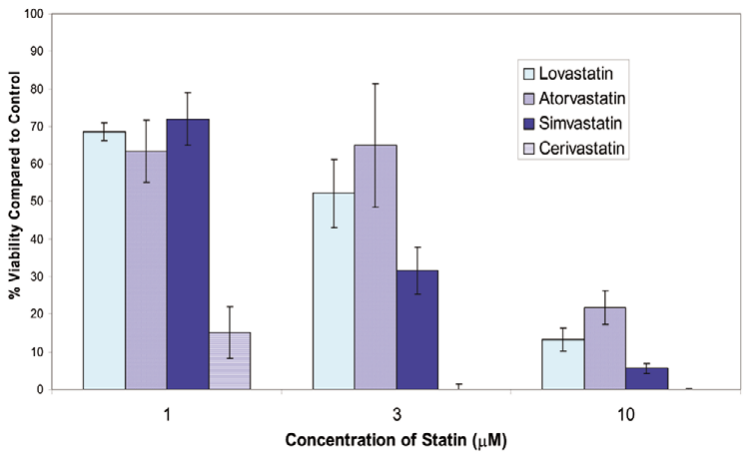
Statins decrease the viability of synovial fibroblasts in a concentration-dependent manner. Synovial fibroblasts from a patient with rheumatoid arthritis were cultured for 96 hours in the presence of 1, 3 or 10 μM lovastatin, atorvastatin, simvastatin or cerivastatin. XTT assays were then performed on quadruplicate wells to determine the percentage cellular viability of statin-treated cultures in comparison with those treated with the vector. Results are representative of two experiments.

**Figure 2:**
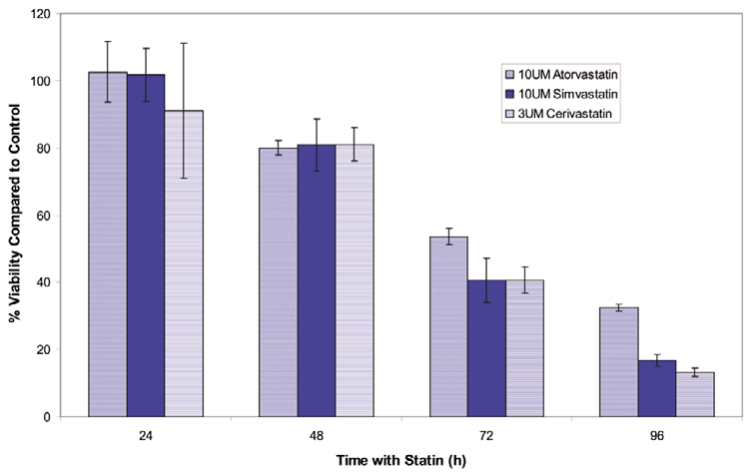
Statins decrease the viability of synovial fibroblasts in a time-dependent manner. Synovial fibroblasts were cultured for 24, 48, 72 and 96 hours in the presence of 10 μM atorvastatin, 10 μM simvastatin or 3 μM cerivastatin. XTT assays were then performed on quadruplicate wells to determine the percentage cellular viability of statin-treated cultures in comparison with those treated with the vector under identical conditions. Results are representative of two experiments.

**Figure 3:**
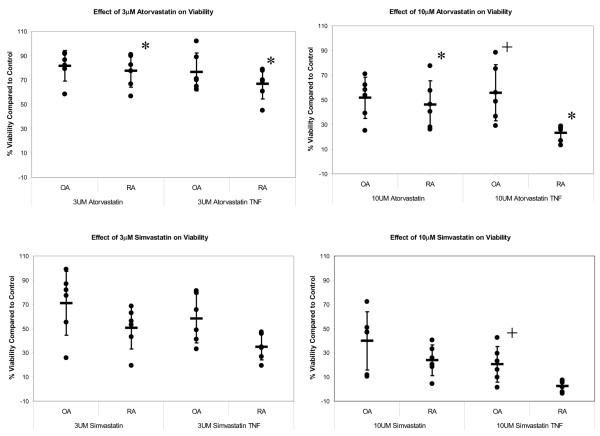
Statins are most effective at inhibiting the viability of TNF-α-stimulated RA synovial fibroblasts. Synovial fibroblasts derived from patients with rheumatoid arthritis (RA) (*n *= 6) and osteoarthritis (OA; *n *= 6) were stimulated or not with 10 ng/ml tumor necrosis factor-α (TNF-α) for 24 hours before the addition of 3 or 10 μM atorvastatin or simvastatin. They were then maintained for a further 96 hours in the presence or absence of TNF-α and statin. XTT assays were performed on quadruplicate wells. The percentage cellular viability was determined by comparing statin-treated cultures with non-treated cultures in either the presence or absence of TNF-α as appropriate. Means and standard deviations are indicated by solid bars. *p < 0.05 for atorvastatin-treated versus simvastatin-treated RA fibroblasts. ^+^*p *< 0.05 for RA versus OA TNF-α-stimulated fibroblasts.

To determine whether statins influenced the viability of RA synovial fibroblasts, RA synovial fibroblasts were cultured in the presence of increasing concentrations of statins ranging from 1 to 10 μM. After 96 hours, cell viability was determined by the XTT assay. The results showed that all of the statins led to reduced viability of RA synovial fibroblasts in a concentration-dependent manner (Figure 1). However, it is noteworthy that simvastatin and cerivastatin reduced FLS viability to a greater extent than lovastatin or atorvastatin. Time-course experiments with atorvastatin (10 μM), simvastatin (10 μM) and cerivastatin (3 μM) revealed that reduced viability occurred in a time-dependent manner (Figure 2). We chose to compare the effects of atorvastatin and simvastatin because they had exhibited different degrees of potency, both on RA synovial fibroblast viability in our preliminary studies and on the CIA mouse model [14]. As shown in Figure 3, in comparison with non-treated cells, simvastatin exhibited a greater effect on the viability of RA synovial fibroblasts (*n *= 6) than atorvastatin both at 3 μM (51 ± 18% versus 77 ± 13% viability, respectively; *p *= 0.014) and at 10 μM (24 ± 13% versus 46 ± 19%, respectively; *p *= 0.04). The effects of the statins were also examined on OA synovial fibroblasts (*n *= 6). Although both simvastatin and atorvastatin reduced the viability of OA synovial fibroblasts, although to a smaller extent than that of RA synovial fibroblasts, no significant differences were observed between the statins at either concentration.

### Statins decrease the viability of TNF-α-stimulated synovial fibroblasts

Because TNF-α is an important driver of RA pathology, we investigated the effects of statins on TNF-α-stimulated proliferation of synovial fibroblasts. Preliminary time-course experiments ranging from 24 to 96 hours (not shown) revealed that similarly to non-treated synovial fibroblasts, those pretreated with TNF-α exhibited decreased viability in a time-dependent manner. As depicted in Figure 3, both statins were able to decrease the viability of TNF-α-stimulated RA synovial fibroblasts (*n *= 6). In TNF-α-stimulated RA synovial fibroblasts, simvastatin caused a marked reduction in synovial fibroblast viability compared with atorvastatin both at 3 μM (35 ± 11% versus 67 ± 13%, respectively; *p *= 0.0008) and at 10 μM (2 ± 5% versus 23 ± 6%, respectively; *p *= 8 × 10^-5^). Reduced RA synovial fibroblast viability was observed in TNF-α-stimulated cells compared with unstimulated cells at 10 μM for both simvastatin (*p *= 0.003) and atorvastatin (*p *= 0.02). As with unstimulated OA synovial fibroblasts, simvastatin reduced the viability of TNF-α-stimulated OA synovial fibroblasts (*n *= 6) to a greater extent than atorvastatin, but no statistically significant differences were observed at 3 μM. In contrast with RA synovial fibroblasts, there were no differences between TNF-α-stimulated and unstimulated OA synovial fibroblasts with either statin at 3 μM or 10 μM. There was a significant difference in reduction in viability between TNF-α-stimulated RA synovial fibroblasts compared with OA synovial fibroblasts at 10 μM, both with simvastatin (*p *= 0.02) and with atorvastatin (*p *= 0.007). These differences were not explained by differences in the degree of stimulation of OA and RA synovial fibroblasts to TNF-α (*p *= 0.72 for the atorvastatin control; *p *= 0.33 for the simvastatin control). Taken together, these results show that although TNF-α stimulates OA and RA synovial fibroblasts equally, RA synovial fibroblasts show a further decrease in viability in the presence of statins.

### Geranylgeranylpyrophosphate restores the viability of RA synovial fibroblasts in the presence of statins

**Figure 4:**
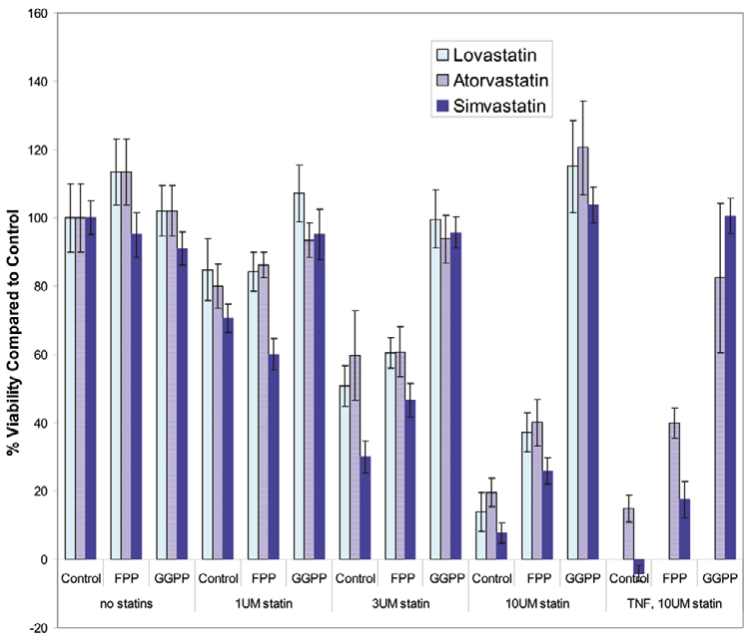
Geranylgeranylpyrophosphate (GGPP) restores the viability of synovial fibroblasts in the presence of statins. Synovial fibroblasts were cultured for 96 hours in the presence of 1, 3 or 10 μM lovastatin, atorvastatin or simvastatin supplemented with 5 μM farnesylpyrophosphate (FPP) or 5 μM GGPP, or neither FPP nor GGPP (control). XTT assays were performed on quadruplicate wells to determine the percentage cellular viability of treated in comparison with non-treated cultures. Each treatment was performed on two different synovial fibroblast lines. A representative experiment is shown. In a separate experiment, synovial fibroblasts were pretreated with 10 ng/ml tumor necrosis factor-α for 24 hours before the addition of 10 μM atorvastatin or simvastatin and 5 μM FPP or 5 μM GGPP. They were cultured for a further 96 hours before an XTT assay on quadruplicate wells.

The decrease in cellular viability caused by statins has been attributed to a lowering of intracellular FPP and GGPP concentrations [25]. This concept was examined in FLS by evaluating the ability of exogenously added FPP and GGPP in the presence of statins to rescue their viability. Our results demonstrated that the statin-induced reduction in RA synovial fibroblast viability could be partly abrogated with 5 μM FPP and completely abrogated with 5 μM GGPP (Figure 4). The complete restoration of viability with GGPP suggests that the inhibition of a geranylgeranylated protein in RA synovial fibroblasts accounts for the reduced viability in the presence of statins.

### Geranylgeranylated proteins are redistributed to the cytoplasm in the presence of statins

**Figure 5:**
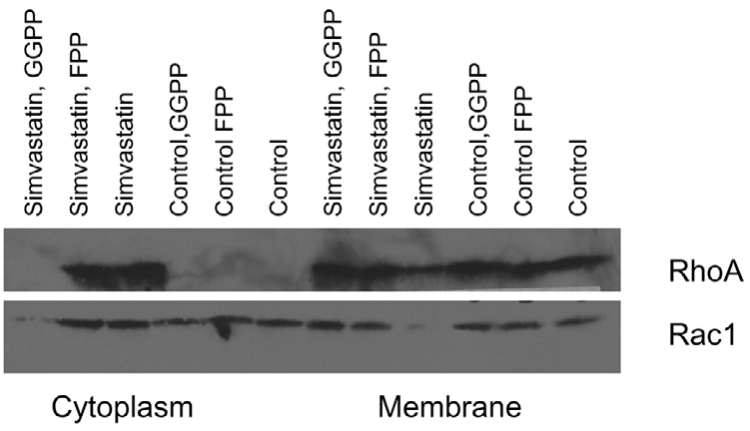
Geranylgeranylated proteins are redistributed to the cytoplasm in the presence of statin. Synovial fibroblasts were cultured for 72 hours in the presence of 3 μM simvastatin supplemented or not with 5 μM farnesylpyrophosphate (FPP) or 5 μM geranylgeranylpyrophosphate (GGPP). Phase separation of proteins was achieved by partitioning with Triton X-114. The entire membrane and cytoplasmic fractions were subjected to PAGE on a 12% gel. Blots were probed with mouse anti-human RhoA or Rac1 followed by goat anti-mouse IgG conjugated to horseradish peroxidase secondary antibody, and were revealed by enhanced chemiluminescence.

To explore the mechanism by which the inhibition of geranylgeranylation leads to decreased RA synovial fibroblast viability, proteins that normally associate with the cell membrane as a consequence of geranylgeranylation were examined for redistribution into the cytoplasm in the presence of statins. Potential candidates for such redistribution are geranylgeranylated members of the Rho subfamily of the Ras superfamily. Members of the Rho family control several cellular processes including transcriptional regulation, cell-cycle progression, cell adhesion and apoptosis. Two geranylgeranylated members of the Rho family that have been implicated in cell survival are RhoA and Rac1. With the use of Triton X-114 partitioning, we demonstrated a decrease in membrane-associated RhoA and Rac1 and an increase in cytoplasm-associated RhoA and Rac1 in the presence of simvastatin (Figure 5). The inclusion of geranylgeranylpyrophosphate in the culture medium restored RhoA and Rac1 to the membrane. The results suggest that statins reduce RA synovial fibroblast viability by affecting the normal cellular distribution of geranylgeranylated proteins such as RhoA and Rac1.

### Reduced RA synovial fibroblast viability with statins results from apoptosis

**Figure 6:**
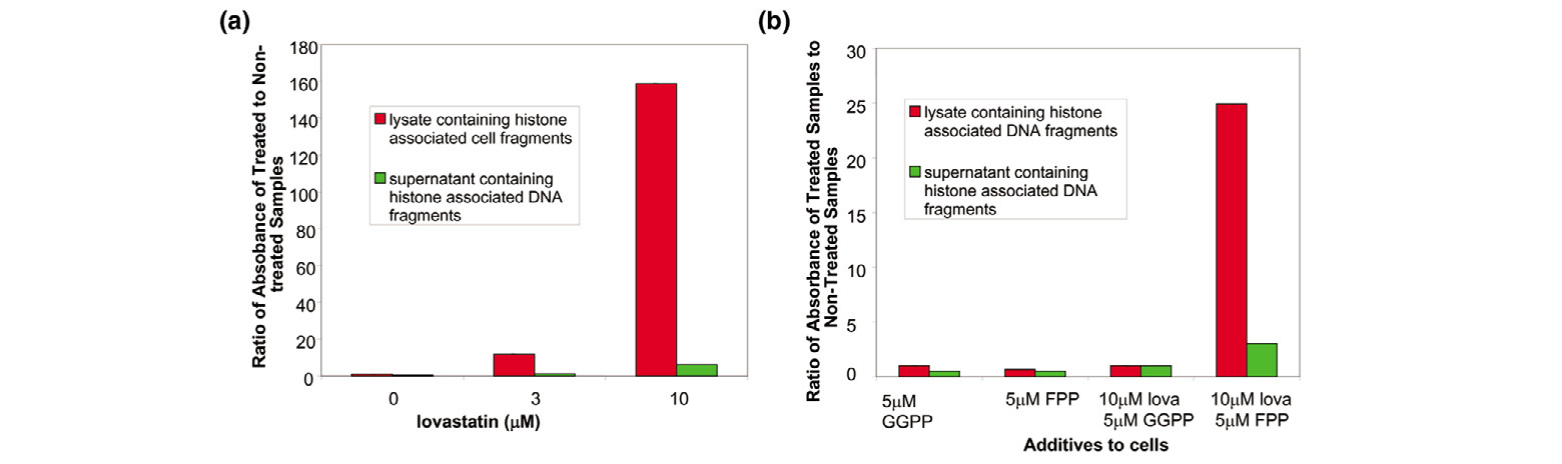
Cellular lysates of synovial fibroblasts cultured in lovastatin contain histone-bound DNA fragments. Cell lysates and corresponding culture supernatants were assayed by Cell Death Detection ELISA^PLUS ^for the presence of histone-bound DNA fragments. **(a)** Synovial fibroblasts were cultured for 72 hours in the presence of 0, 3 or 10 μM lovastatin. The dark bars represent fragments present in the cell lysate indicative of apoptosis; the light bars represent fragments present in the supernatant. Shown is a representative of three different experiments. **(b)** Synovial fibroblasts were cultured for 72 hours in 10 μM lovastatin supplemented or not with 5 μM farnesylpyrophosphate (FPP) or 5 μM geranylgeranylpyrophosphate (GGPP).

**Figure 7:**
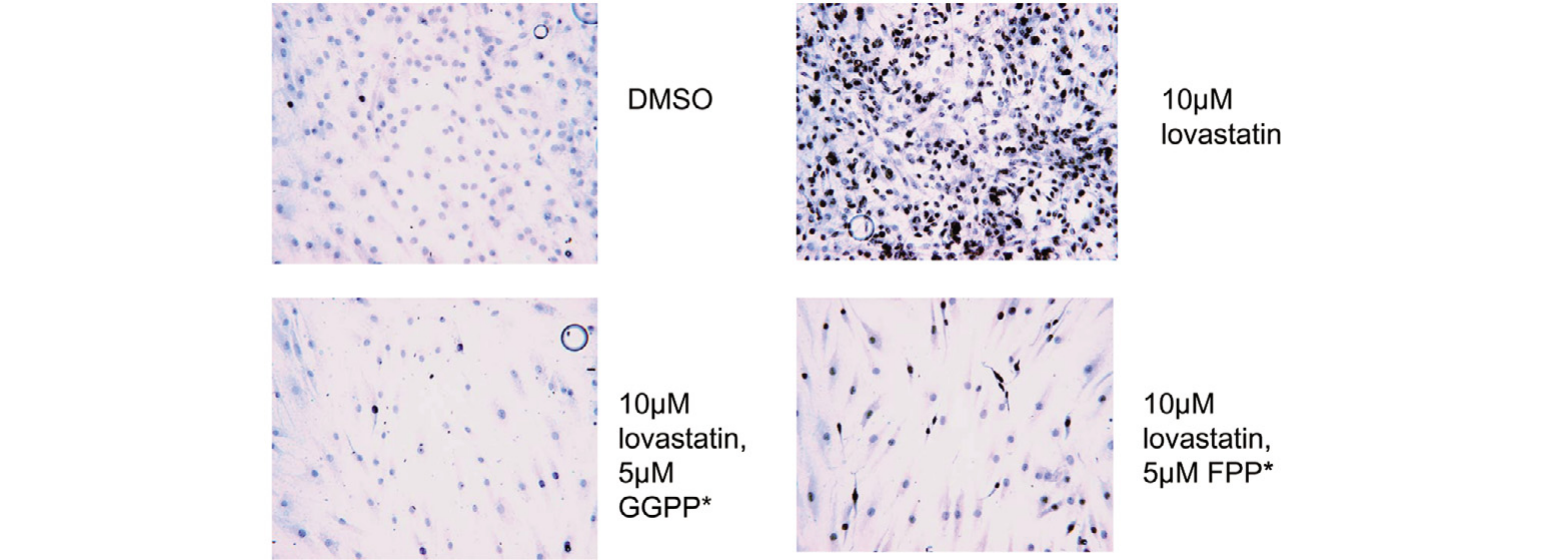
TUNEL assay confirms the presence of DNA fragments in synovial fibroblasts treated with lovastatin. DNA fragments in synovial fibroblasts treated or not with 10 μM lovastatin for 72 hours were detected by a TdT-FragEL DNA fragmentation kit. After TdT-mediated dUTP nick end labelling (TUNEL) staining, cells were counterstained with hematoxylin. TUNEL-positive cells have dark nuclear staining, whereas the nuclei of TUNEL negative cells stain blue. Asterisk, 5 μM geranylgeranylpyrophosphate (GGPP) or 5 μM farnesylpyrophosphate (FPP) were included with the lovastatin.

To determine whether apoptosis accounts for the decrease in RA synovial fibroblast viability we measured histone-bound DNA fragments in cell lysates and used a TUNEL assay to measure DNA fragments. Lysates prepared from RA synovial fibroblasts grown in increasing concentrations of lovastatin contained increasing amounts of histone bound DNA fragments, suggesting the induction of apoptosis (Figure 6a). Histone-bound DNA fragments decreased about sixfold with the addition of 5 μM FPP and returned to baseline with the addition of 5 μM GGPP (Figure 6b). Similar results were obtained with the TUNEL assay (Figure 7). Taken together, these results suggest that the reduced viability of synovial fibroblasts induced by statins results from apoptosis brought about primarily by the decrease in membrane-associated geranylgeranylated proteins.

### The Akt survival pathway is inhibited by statins in RA synovial fibroblasts

**Figure 8:**
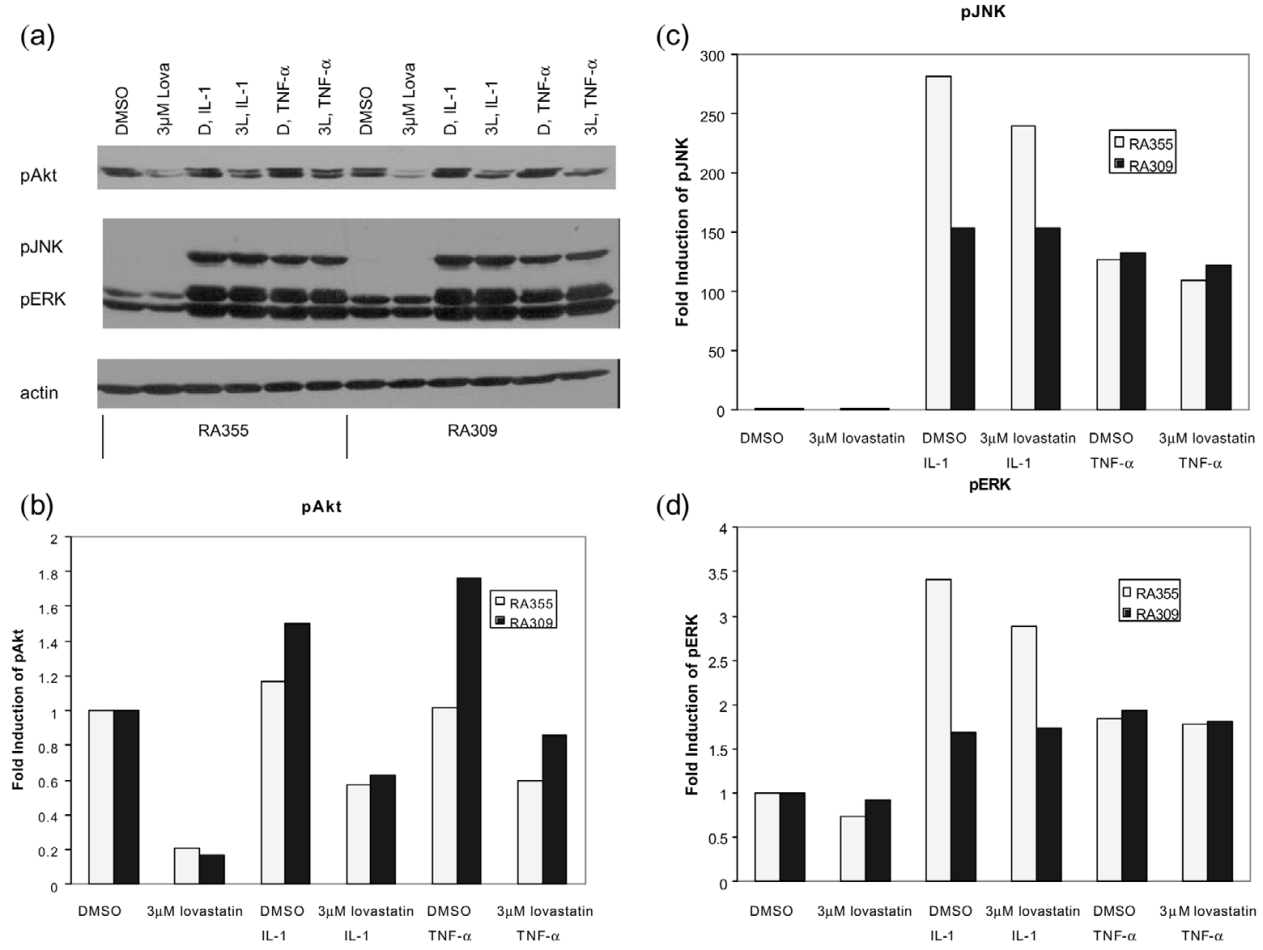
The steady-state and activated Akt pathway is affected by lovastatin. Rheumatoid arthritis synovial fibroblasts (2 lines shown) were cultured in the presence of 3 μM lovastatin or DMSO (solvent control) for 48 hours. They were then treated with 2 ng/ml IL-1 or 10 ng/ml tumor necrosis factor-α (TNF-α) for 15 minutes. **(a)** Cellular lysates were prepared, subjected to 12% PAGE and analysed by immunoblotting for pAkt, pJNK, pERK and actin. D, dimethylsulfoxide (DMSO); 3L, 3 μM lovastatin; D, IL-1, cells cultured with DMSO then induced with IL-1; D, TNF, cells cultured with DMSO then induced with TNF-α; 3L, IL-1, cells cultured with 3 μM lovastatin then induced with IL-1; 3L, TNF, cells cultured with 3 μM lovastatin then induced with TNF-α **(b-d)** Bands were quantified by UN-SCAN-IT and corrected for loading by actin (b) pAkt, (c) pJNK, (d) pERK.

We next examined intracellular signaling pathways implicated in RA synovial fibroblast survival. Lovastatin decreased the steady-state pAkt levels at least threefold, and TNF-α or IL-1 signalling through pAkt was also prevented (Figure 8). In contrast, neither the steady-state levels nor the TNF-α or IL-1β induction of pJNK or pERK were affected by culturing RA synovial fibroblasts with 3 μM lovastatin. This suggests that the Akt survival pathway is inhibited by statins in RA synovial fibroblasts.

### siRNA-directed inhibition of Rac1 reduces RA synovial fibroblast viability and pAkt levels

**Figure 9:**
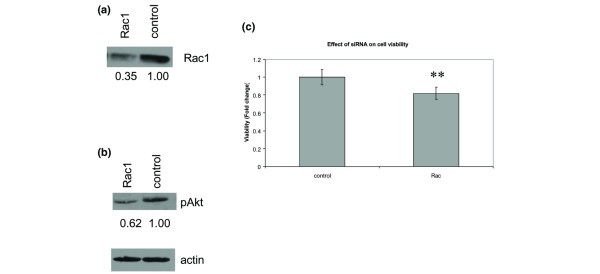
siRNA-directed inhibition of Rac1 reduces synovial fibroblast viability and pAkt levels. Synovial fibroblasts were transfected with siRNA against Rac1 or a non-silencing RNA as control, and cultured for 96 hours at which point XTT assays were performed to measure cell viability and lysates were prepared from wells that had been transfected in parallel. **(a, b)** Lysates were subjected to immunoblotting and probed with anti-Rac **(a)** or anti-pAkt or actin **(b)** for normalization. **(c)** Effect of siRNA on cell viability. ** *p *< 0.01 for viability of synovial fibroblasts transfected with non-siRNA control versus Rac1 siRNA. Shown is a representative of three independent experiments. siRNA transfection efficiency was greater than 95%.

To determine the relationship between the decrease in geranylgeranylated proteins and pAkt levels, we used siRNA against Rac1. Rac1 was selected because the expression of a dominant-negative form of Rac1 has been shown to sensitize cells to TNF-α-mediated apoptosis [26,27]. RA synovial fibroblasts transfected with siRNA against Rac1 exhibited a decrease in both Rac1 and pAkt protein levels in comparison with a control siRNA against no known protein coding sequence (Figure 9a,b). Furthermore, synovial fibroblasts transfected with siRNA against Rac1 exhibited decreased viability as determined in an XTT assay (*n *= 3; *p *< 0.01; Figure 9c). These results suggest that the inhibition of Rac1 affects pAkt levels and that Rac1 inhibition influences RA synovial fibroblast viability.

## Discussion

We have shown that statins decrease RA synovial fibroblast viability in a concentration-dependent and time-dependent manner. Simvastatin is significantly more effective than atorvastatin at inhibiting RA synovial fibroblast survival. Similar differences in the efficacy of different statins have been reported previously in tumor-specific apoptosis [28]. The difference between simvastatin and atorvastatin was even greater in cells preactivated with TNF-α. Although both statins also decreased OA synovial fibroblast survival, there was no significant difference between them in their ability to reduce survival. Moreover, they were not significantly more effective in reducing OA synovial fibroblast survival in the presence of TNF-α. Taken together, these data suggest that in RA synovial fibroblasts, statins are inhibiting TNF-α regulated synovial fibroblast survival, enabling TNF-α to function as an apoptotic signal rather than as a survival signal. Our results demonstrated that all of the statins examined seem to exert their effects in a similar manner through the inhibition of a geranylgeranyl pyrophosphate intermediate, resulting in a loss of membrane-associated geranylgeranylated proteins and a subsequent reduction in viability of synovial fibroblasts. All statins consist of a dihydroxyheptanoic group that mimics mevalonate, but they have different chemical substituents elsewhere on the molecule that can lead to differences in their efficacy [29]. It is therefore possible that differences in the efficacies of the statins on RA synovial fibroblast viability result from differences in the molecules other than the dihydroxyheptanoic group. These differences account for differences in the bioavailability of the statins and the active metabolites generated by the cytochrome P450 family of enzymes [30].

As far as we are aware, this is the first report to implicate geranylgeranylated proteins in synovial fibroblast viability. The presence of this pathway in both OA and RA synovial fibroblasts suggests that it is a constitutive pathway in synovial fibroblast biology. However, the inhibition by GGPP of the statin effect on TNF-α-stimulated RA synovial fibroblasts suggests that in RA synovial fibroblasts, TNF-α is signaling through the same pathway that is required for their overall viability and that this pathway requires a functional geranylgeranylated protein. This pathway differs in RA synovial fibroblasts compared with OA synovial fibroblasts, as demonstrated by the significant difference in the effect of statins between TNF-α-stimulated and non-stimulated RA synovial fibroblasts not seen with OA synovial fibroblasts.

The data in this study also show that the statins affect the Akt pathway in RA synovial fibroblasts. Previous studies have demonstrated that synovial tissue obtained from patients with RA expresses higher levels of pAkt than that derived from patients with OA [31]. It was also demonstrated that elevated pAkt levels account for the anti-apoptotic response of synovial fibroblasts to both TNF-α and transforming growth factor-β, suggesting that elevated pAkt might contribute to the establishment of synovial hyperplasia observed in patients with RA [31,32]. The phosphoinositide 3-kinase/Akt pathway is being increasingly recognized as playing a major role in synovial fibroblasts. It is used in response to TRAIL (TNF-related apoptosis-inducing ligand) [33], the transmission of IL-18 signals leading to VCAM (vascular cell adhesion molecule) expression [34] and the production of IL-6 and IL-8 stimulated by IL-17 [35]. The results presented in the present study lend further support to the importance of elevated pAkt levels to synovial fibroblast survival and suggest that statins might have a beneficial role in reducing the aberrant pAkt levels in patients with RA.

Experiments with siRNA technology implicated Rac1 in synovial fibroblast survival. Additional experiments are currently under way to determine whether other geranylgeranylated proteins contribute to synovial fibroblast survival. We also demonstrated decreased levels of pAkt in these cells. These data suggest that a survival signal is propagated in synovial fibroblasts via a membrane-associated geranylgeranylated protein, Rac1 with subsequent pAkt activation. These data are consistent with other studies of Rac1-regulated Akt resulting in an anti-apoptotic signal [36-38].

The results of this study have several implications for the therapeutic use of statins in patients with RA. In the TARA study of patients with RA, atorvastatin significantly improved the swollen joint count, C-reactive protein, erythrocyte sedimentation rate, fibrinogen, soluble intercellular adhesion molecule 1 (sICAM1) and IL-6 [15]. Because synovial fibroblasts synthesize sICAM1 and IL-6 it is possible that the decreased level observed results in part from atorvastatin-driven decreased synovial fibroblast viability. If this were so, our studies suggest that simvastatin might be more effective than atorvastatin in patients with RA. Previous investigation demonstrated that the administration of simvastatin in a CIA model resulted in decreased proliferation of T cells, but it was not reported whether this resulted from apoptosis [13]. There is the possibility that the effect of statins might be cell type specific, a concept supported by differential effects of statins on human lung fibroblasts, human atrial myofibroblasts, and lymphoma tumour cells compared with human pancreatic islets and microglia [19,39-42].

Our results show that the pathway affected by statins in RA synovial fibroblasts is also a TNF-α anti-apoptotic pathway. We are currently dissecting these pathways with a view to discerning new therapeutic targets for RA.

## Conclusion

Statins attenuate the insertion of geranylgeranylated proteins into the plasma membrane with a resultant decrease in viability and increased apoptosis of synovial fibroblasts. A significant difference in the efficacy of simvastatin from that of atorvastatin was observed in both activated and non-activated RA synovial fibroblasts but not OA synovial fibroblasts, suggesting a fundamental difference in their intracellular signaling pathways. The intracellular redistribution of geranylgeranyl proteins resulting from treatment with statin is correlated with decreased activation of Akt, a pathway previously identified as being anti-apoptotic in synovial fibroblasts. These results suggest that statins, particularly simvastatin, might have a beneficial role in reducing aberrant pAkt levels in the synovial fibroblasts of patients with RA, with the resultant decrease in synovial hyperplasia.

## Abbreviations

CIA = collagen-induced arthritis; DMSO = dimethylsulfoxide; FLS = fibroblast-like synoviocytes; FPP = farnesylpyrophosphate; GGPP = geranylgeranylpyrophosphate; HMG = hydroxymethylglutaryl; IL = interleukin; OA = osteoarthritis; PBS = phosphate-buffered saline; RA = rheumatoid arthritis; siRNA = short interfering RNA; TNF-α = tumor necrosis factor-α; XTT = sodium 3-(1-(phenylamino-carbonyl)-3,4-tetrazolium)-bis (4-methoxy-6-nitro) benzenesulfonic acid hydrate; TUNEL = TdT-mediated dUTP nick end labelling.

## Competing interests

The authors declare that they have no competing interests.

## Authors' contributions

AC participated in the design of the study, performed the experiments and participated in the writing of the manuscript. SB and AN participated in the design of the study and the writing of the manuscript. EK participated in the design of the study, the analysis of data and the writing of the manuscript. All authors read and approved the final manuscript.
